# Potential biomarkers of acute myocardial infarction based on weighted gene co-expression network analysis

**DOI:** 10.1186/s12938-019-0625-6

**Published:** 2019-01-25

**Authors:** Zhihua Liu, Chenguang Ma, Junhua Gu, Ming Yu

**Affiliations:** 1 Shenzhen Institutes of Advanced Technology, Chinese Academy of Sciences, Shenzhen, 518055 China; 2 Tsinghua University, Beijing, 100084 China; 3 Beijing Yuqiu Medical Research Institute, Beijing, 100022 China; 4 Shenzhen Yuqiu Biological Big Data Research Institute, Shenzhen, 518033 China; 5 Nanjing Yuqiu Biotechnology Co., Ltd., Nanjing, 210009 China; 6 Hebei University of Technology, Tianjin, 300130 China

**Keywords:** Acute myocardial infarction, Weighted gene co-expression network analysis, Gene ontology, Functional enrichment analysis

## Abstract

**Background:**

Acute myocardial infarction (AMI) is the common cause of mortality in developed countries. The feasibility of whole-genome gene expression analysis to identify outcome-related genes and dysregulated pathways remains unknown. Molecular marker such as *BNP*, *CRP* and other serum inflammatory markers have got the notice at this point. However, these biomarkers exhibit elevated levels in patients with thyroid disease, renal failure and congestive heart failure. In this study, three groups of microarray data sets (GES66360, GSE48060, GSE29532) were collected from GEO, a total of 99, 52 and 55 samples, respectively. Weighted gene co-expression network analysis (WGCNA) was performed to obtain a classifier which composed of related genes that best characterize the AMI.

**Results:**

Here, this study obtained three groups of microarray data sets (GES66360, GSE48060, GSE29532) on AMI blood samples, a total of 99, 52 and 24 samples, respectively. In all, 4672 genes, 3185 genes, 3660 genes were identified in GSE66360, GSE48060, GSE60993 modules, respectively. We preformed WGCNA, GO and KEGG pathway enrichment analysis on these three data sets, finding function enrichment of the differential expression gene on inflammation and immune response. Transcriptome analysis were performed in AMI patients at four time points compared to CAD patients with no history of MI, to determine gene expression profiles and their possible changes during the recovery from myocardial infarction.

**Conclusions:**

The results suggested that three overlapping genes (*FGFBP2*, *GFOD1* and *MLC1*) between two modules could be a potential use of gene biomarkers for the diagnose of AMI.

## Introduction

Despite significant advances in revascularization strategies, pharmacotherapy, cardiac rehabilitation algorithms and organ transplantation, cardiovascular diseases, including acute myocardial infarction (AMI), remains the leading cause of death in developed countries [1]. The classic risk factors such as smoking, high serum cholesterol, hypertension and diabetes mellitus that can partly predict in disease prevention and outcomes, but is not sufficient to fully provide acute diagnosis [2]. An important challenge to implementing such strategies is the limited predictive value of current risk forecast models. This “detection gap” is illustrated by the observation that patients with coronary heart disease often lack conventional risk factors, up to 20% of patients have no traditional risk factors, and 40% have only one [3, 4]. Currently more attention is given to nontraditional as well as genetics risk factors, thus, improved strategies for controlling the development of AMI, reducing the mortality rate and improving prognosis are a public health priority.

Molecular marker such as *BNP*, *CRP* and other serum inflammatory markers have got the notice at this point, however, these biomarkers exhibit elevated levels also in patients with thyroid disease, renal failure and congestive heart failure [5], mandating the search for novel more sensitive risk markers to improve the selection of individuals for preventative strategies. Such biomarkers must meet the ability to identify individuals at risk, stability of results when repeated and therapeutic impact with early intervention, and motivate hypotheses for cell-based therapeutic targets to control the pathological process of AMI.

Invasive coronary angiography is the “gold standard” for detecting AMI. However, this is costly, and can pose risk to the patient. Genome-wide expression analysis using microarray is an extensively used strategy for the detection of new biomarkers for diagnosis, prediction of disease severity and identification of novel drug targets [6, 7]. In this study, our main goal were: (1) to establish alterations in gene expression patterns in a peripheral blood cell model from patients with AMI and through a follow up; (2) to identify distinct biomarkers that correlate with AMI and development.

For co-expression data analysis, three groups of microarray data sets (GES66360, GSE48060, GSE29532) were downloaded from GEO, a total of 99, 52 and 55 samples, respectively. We proposed a simple analysis method used for finding clusters (modules) of highly correlated genes, called “weighted correlation network analysis” (WGCNA) to obtain a classifier which composed of related genes that best characterize the AMI. In addition, we performed gene ontology (GO) and Kyoto encyclopaedia of genes and genomes (KEGG) pathway analyses of the significant clusters.

## Method

### Data

The microarray expression data sets (GES66360, GSE48060, GSE60993) were downloaded from the GEO database. This study samples consisted of first-time AMI patients and healthy controls with a normal echocardiogram. The data set GSE60993 was collected from peripheral blood containing 17 patients and 7 control samples, using the Illumina HumanWG-6 v3.0 expression beadchip. The other two data sets GSE48060 and GES66360 were both used the Affymetrix Human Genome U133 Plus 2.0 Array platform, including 49 patients versus 50 controls collected from circulating endothelial cells, and 31 patients versus 21 controls collected from peripheral blood, respectively.

The aim of our second microarray cohort analysis was to find expression of genes linked specially with the follow-up of AMI,to exclude genes linked with coronary artery disease (CAD). Here, we chose the control group patients with stable CAD, not the healthy controls.

### Differential expression

Before the differential expression analysis, we performed the step of preprocessing. If a gene had more than one probe site, we averaged the values of the probe sites.

After the preprocessing, we performed variance analysis between the AMI patient group and the healthy control group for the three data sets. And the statistical significance threshold level for differential expression genes was P < 0.05. So we identified 4672 genes, 3185 genes, 1167 genes in GES66360, GSE48060, GSE29532 data sets, respectively.

### Weighted gene co-expression network analysis (WGCNA)

WGCNA was used the scale free topology criterion to construct the gene co-expression network. The differential expression genes of the three data sets were independently analyzed using the WGCNA method. First, for each set of genes, a weighted correlation network was constructed by computing a pair-wise correlation matrix. We arrange the correlations into a matrix: Let *p*_*ij*_ be the correlation between variables *i* and *j*. Place *p*_*ij*_ into positions (*i*, *j*) and (*j*, *i*) of the correlation matrix. The absolute value of the correlation is used as an unsigned co-expression similarity measure (Eq. 1).

1 $$ p_{ij}^{unsigned} = \left| {cor\left( {x_{i} ,\;x_{j} } \right)} \right|. $$

Next, an adjacency matrix was constructed by raising the correlation matrix to the power of 8. Based on the adjacency matrix, the topological overlap measure of each pair of genes was calculated. Then, we performed average linkage hierarchical clustering on the topological overlap, and used the Dynamic Hybrid Tree Cut algorithm to cut the clustering tree. To obtain large and distinct modules, we set the minimum modules size to 30 genes and the minimum height for merging modules at 0.2.

### Functional enrichment analysis

For each module genes, GO and Pathway Functional enrichment analysis was assessed using hypergeometric test. The calculating formula is:

2 $$ {\text{P}} = 1 - \mathop \sum \limits_{{{\text{i}} = 0}}^{{{\text{m}} - 1}} \frac{{\left( {\begin{array}{*{20}c} {\text{M}} \\ {\text{i}} \\ \end{array} } \right)\left( {\begin{array}{*{20}c} {{\text{N}} - {\text{M}}} \\ {{\text{n}} - {\text{i}}} \\ \end{array} } \right)}}{{\left( {\begin{array}{*{20}c} {\text{N}} \\ {\text{n}} \\ \end{array} } \right)}} . $$

In Eq. (2), *N* is the number of all genes with annotation; n is the number of module genes in *N*; *M* is the number of all genes that are annotated to the certain terms; *m* is the number of module genes in *M*. The calculated *P* value goes through Bonferroni Correction, taking corrected *P* value 0.05 as a threshold.

## Results

### Microarray differentially expressed gene between AMI and healthy controls

**Fig. 1:**
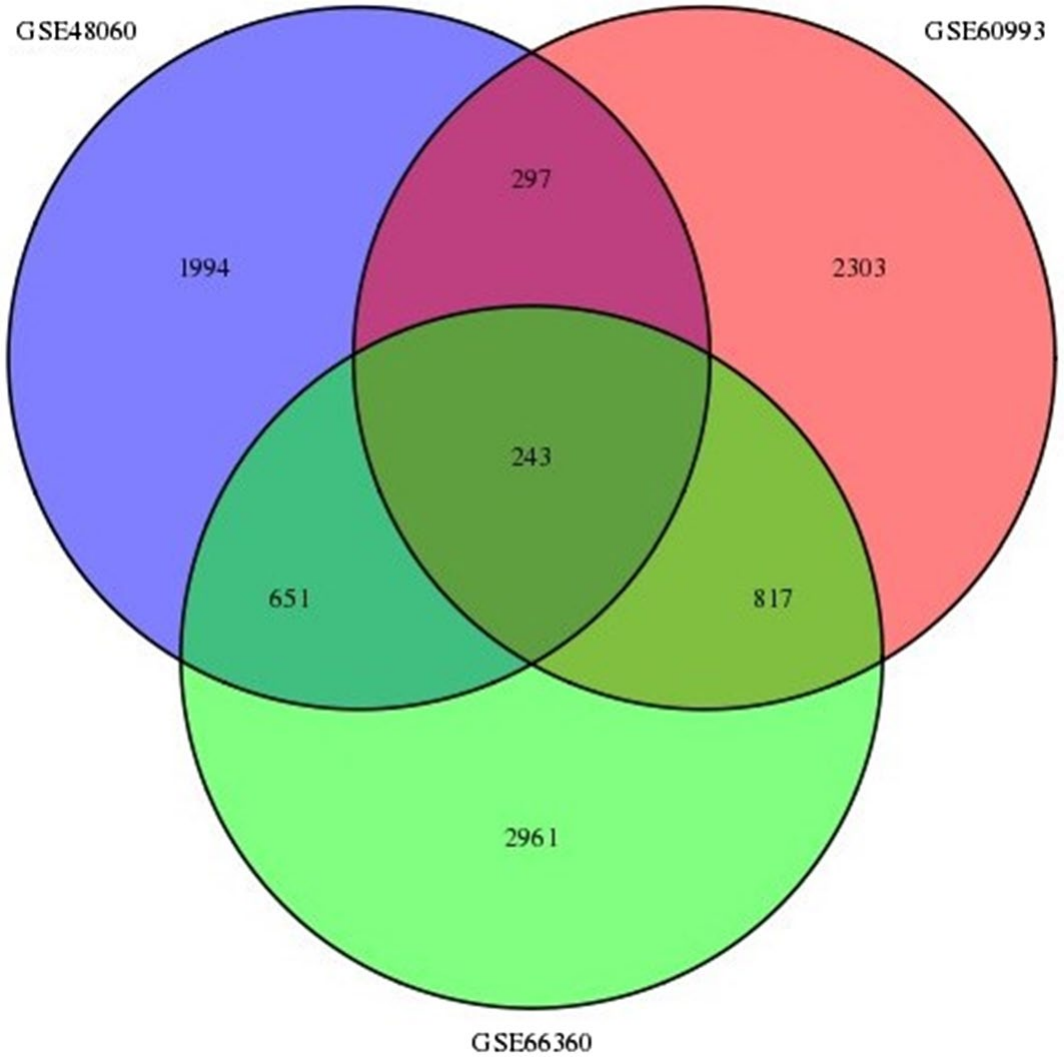
A venn diagram describing the overlap between genes differentially expressed in three cases

We first selected the global different genes in the organization of gene transcriptome between AMI and control groups. We identify 4672 genes, 3185 genes, 3660 genes in GSE66360, GSE48060, GSE60993 groups, respectively (Fig. 1). The significant expression changes could be showed between AMI and control groups. We observed a highly significant overlap between differentially expressed genes in three of cases, supporting the robustness of the data and indicating the AMI-specific expression changes are consistent across these three cases.

Next, we applied WGCNA to integrate the expression differences into a higher order, systems level context. The pathways involved in each module were summarized in Table 1.

**Table 1:** The pathways involved in three cases

Modules	Pathways	Q-value (Q < 0.05)
GSE48060	Natural killer cell mediated cytotoxicity	1.066849e−07
	Graft-versus-host disease	1.956196e−07
	Allograft rejection	1.237023e−05
	Cytokine-cytokine receptor interaction	1.231519e−04
	Type I diabetes mellitus	3.664642e−04
GSE60993	Ribosome	1.315470e−06
	Intestinal immune network for IgA production	8.294981e−05
	Allograft rejection	1.018873e−04
	Hematopoietic cell lineage	3.178231e−04
	NF-kappa B signaling pathway	4.203928e−04
GSE66360	Graft-versus-host disease	7.291560e−18
	Natural killer cell mediated cytotoxicity	1.330539e−15
	Antigen processing and presentation	1.330539e−15
	Cytokine-cytokine receptor interaction	3.994169e−01
	Gap junction	3.994169e−01

We chose the appropriate module which show significant differences (Q < 0.05) and related to the progression of AMI. For these three sets of data, we selected the midnightblue module of GSE66360 group, the pink module of the GSE48060 group, the brown module of the GSE60993 group. Then supervised hierarchical clustering based on the top differentially expressed genes showed distinct clustering of the samples.

**Fig. 2:**
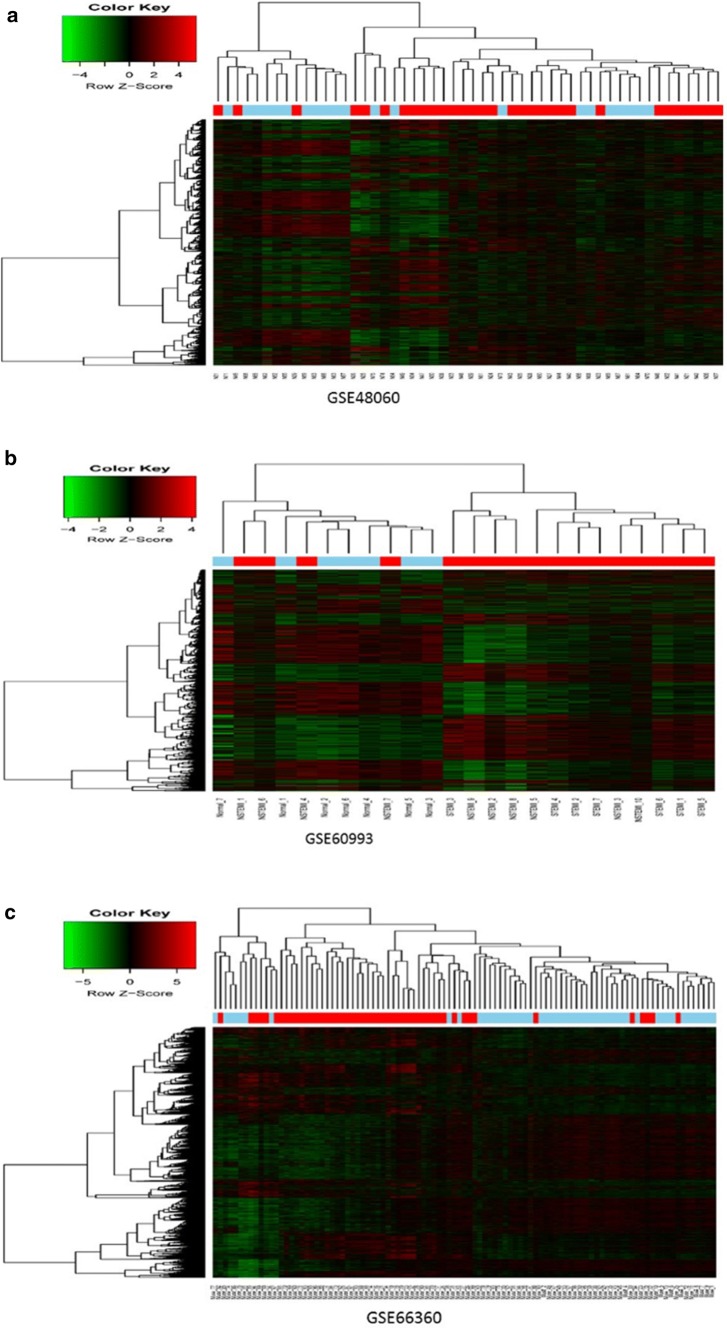
The heat map of modules correspond to GSE48060 (**a**), GSE60993 (**b**), GSE66360 (**c**) differentially expressed between AMI and control samples. Scaled expression values are colour-coded according to the legend on the left. The dendrogram depicts hierarchical clustering of samples and gene expression based on the differentially expressed genes. The top bar indicates the disease status: red, AMI; black, control

Clustering analysis did not identify significant global differences between groups on visual inspection. However, hierarchical cluster analysis showed clustering of gene expression and samples in the GSE66360, GSE48060, GSE60993 group, all three were divided into two distinct parts. Genes involved in the differential module were then subjected to functional annotation and pathway enrichment analysis, the specific results as shown in the Fig. 2a–c. We also observed only three overlapping genes between differentially expressed genes in these three selected modules: *FGFBP2*, *GFOD1*, *MLC1*, which were functionally enriched for revascularization, glycometabolism, Megalencephalic leukoencephalopathy with subcortical cysts pathways.

### Gene expression profiling at different time points following AMI

To determine gene expression profiles and their possible changes during the recovery from myocardial infarction, we performed a transcriptome analysis in AMI patients at four time points compared to CAD patients with no history of MI.

**Fig. 3:**
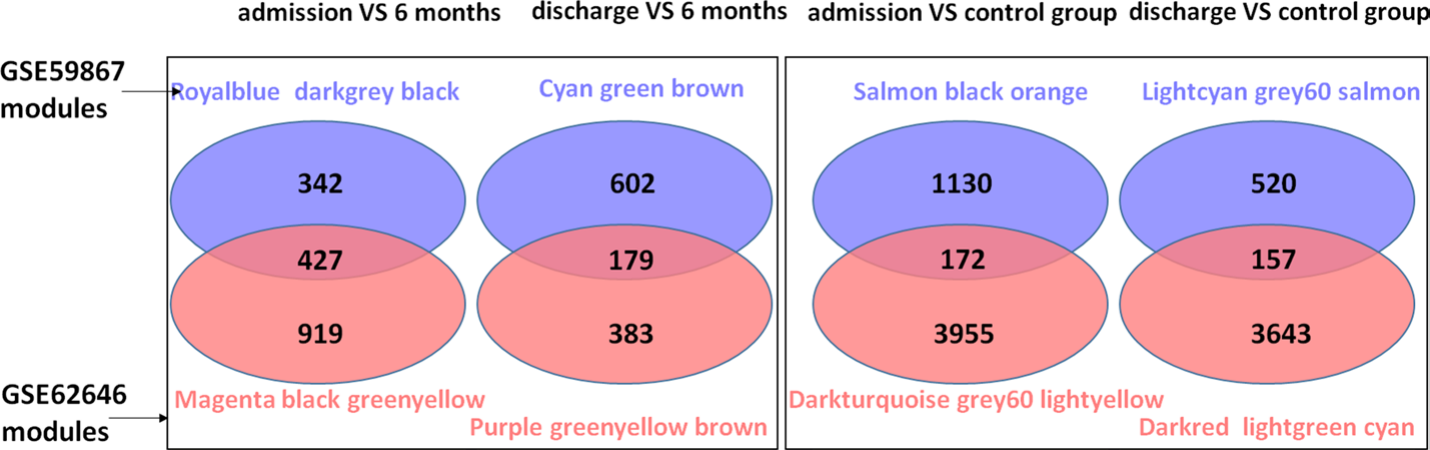
Quantitative assessments GSE59867 and GSE62646 modules at different time points following AMI

A comparison between the samples from the first two time points (admission, discharge after AMI) and samples from the same patients collected 6 months after AMI or control group (patients with a stable coronary artery disease and without a history of myocardial infarction) was performed to identify genes between the comparisons. We also applied WGCNA to identify modules of co-expressed genes with high topological overlap. Finally, module eigengenes are calculated, which provides quantitative assessments of modules for further analysis. We identified a 172 transcripts overlap between the GSE59867 and GSE62646 data set on admission versus the control group (Fig. 3).

In a canonical analysis three most significant pathways were: Natural killer cell mediated cytotoxicity, Graft-versus-host disease, Antigen processing and presentation (Table 2). The expression of 427 genes within 6 months after AMI was changed relative to admission, and pathway analysis of these different expressed transcripts were mainly associated with Oxidative phosphorylation, Huntington’s disease and Parkinson’s disease. On discharge 157 transcripts were differentially expressed compared to control group, and 179 transcripts compared to 6 months after AMI. Notably, the pathway enrichment analysis results showed that admission versus control and discharge versus control group share the similar pathways, the admission versus 6 month and discharge versus 6 month group share the similar pathways.

**Table 2:** The pathways involved in differentially expressed genes compared with different time points following AMI

	Pathways	Q-value (Q < 0.05)
admission versus control group	Natural killer cell mediated cytotoxicity	3.142005e−09
	Graft-versus-host disease	5.779472e−07
	Antigen processing and presentation	2.997803e−03
	Cytosolic DNA-sensing pathway	2.302012e−02
discharge versus control group	Natural killer cell mediated cytotoxicity	8.154634e−10
	Graft-versus-host disease	1.164728e−08
	Antigen processing and presentation	1.657503e−04
	Allograft rejection	2.438676e−02
admission versus 6 months	Oxidative phosphorylation	3.841450e−15
	Huntington’s disease	1.570003e−12
	Parkinson’s disease	2.462576e−12
	Alzheimer’s disease	1.076534e−11
discharge versus 6 months	Parkinson’s disease	4.627948e−16
	Oxidative phosphorylation	4.627948e−16
	Alzheimer’s disease	9.071314e−15
	Huntington’s disease	8.428429e−14

Additionally, we undertook a patient-by-patient analysis of the most differently expressed genes between above four groups, and identified 19 common genes in all patients. The direction of the most significant change was the same although the relative levels of expression differed markedly between patients.

## Discussions

The most important limitation for accurate investigation of the pathophysiology of AMI is the necessity for heart tissue sample [8, 9]. Transcriptome technologies have provided new opportunities to discover disease-specific mechanisms which were distinguishable and may offer diagnostic and prognostic value [10–18]. In this paper, we compared the two transcriptome profiles: AMI patients versus healthy controls, admission, discharge, and 6 months after AMI patients versus with the controls (not the healthy group), with the aims to identify potential cardiac ischemia-related biomarkers and revealed the anticipated modulation networks and pathways that correlate with AMI development.

### AMI patients versus healthy controls

We retained the 243 overlap between differentially expressed genes in AMI and control group in three cases, which were functionally enriched for inflammation, immune response pathways in AMI patients compared to matched, healthy controls. These findings are consistent with previous studies which also demonstrated the increased gene levels for immune response, inflammation and apoptosis pathways, raising the possibility of relationship between myocardial pathological procedure and inflammatory transcriptional changes in circulating cells. Indeed, targeted gene studies by measuring directly the inflammatory mRNA profile of leucocytes in multigene system, supporting the hypothesis that an inflammatory response involving leucocytes contributed to the pathogenesis of myocardial infarction [19]. For another, as in the modulation of leukocyte transcriptional machinery, the analyses of changes in blood leukocyte gene expression patterns reveals that the human blood leukocyte response to transient dysregulation and modulation of translational machinery, providing further evidence that myocardial ischemia results in transcriptional gene expression changes in the peripheral blood [20, 21].

The complexity of the dataset was reduced by lowing non-significant probe sets using WGCNA method, the most significant module was generated in each case. We found only three overlapping genes between the pink module of the GSE48060 group, the midnightblue module of the GSE66360 group and the brown module of the GSE60993 group. These three genes were FGFBP2, GFOD1 and MLC1. This finding implied the AMI pathogeny was associated with an increased expression of genes involved in the intravascular lesions, immunological responses and brain-derived factor regulatory system, suggesting a potential use of FGFBP2, GFOD1 and MLC1 as gene expression biomarkers for very early stages of AMI.

We then selected the co-expression module that showed great significance to analyze biological functions, these analyses associated inflammation and immune response pathway including natural killer cell mediated cytotoxicity, graft-versus-host disease, allograft rejection, antigen processing and presentation and Cytokine-cytokine receptor interaction with pathological changes following ischemic cardiac injury. These findings provide evidence that profiling of circulating cells in patients is capable of identifying distinct, biologically relevant gene expression changes and modulated networks and pathways. It’s worth mentioning that functional analysis of these differential gene sets revealed the enrichment of Type I diabetes mellitus and Autoimmune thyroid disease pathways. The subclinical hypothyroidism (associated with reduced systolic function, an atherogenic lipid profile, diastolic hypertension, and inflammatory condition) and hyperthyroidism (related to a mild decreased of coronary reserve, and an increased risk of supraventricular arrhythmias, hypercoagulable state) have recently been documented as clinical entities with negative effects on the cardiovascular system [22, 23]. Thus, mild forms of thyroid hormones disorders, often leads to the similar changes in cardiac function and gene expression, even small variations of the thyroid hormone within the physiological range may cause the adverse cardiovascular development. This supports the utility of large-scale gene expression analysis of the blood transcriptome at initial diagnosis of AMI for identification of relevant mechanisms of disease.

### Admission, discharge, and 6 months after AMI patients versus with the controls

Numerous studies found significant roles of PBMCs in the systemic and regional inflammatory responses associated with remodeling in AMI [24, 25]. Thus, activation of the PBMCs, which reflects the magnitude of inflammation, could be linked to the progression of AMI patients.

**Fig. 4:**
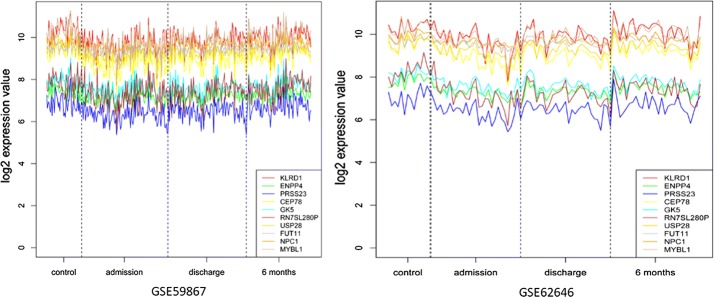
Expression data from microarray experiments in PBMCs—GSE59867 and GSE62646 at different stage. The y-axis represents the log2 normalized intensity of the gene and the x-axis represents analyzed groups

**Fig. 5:**
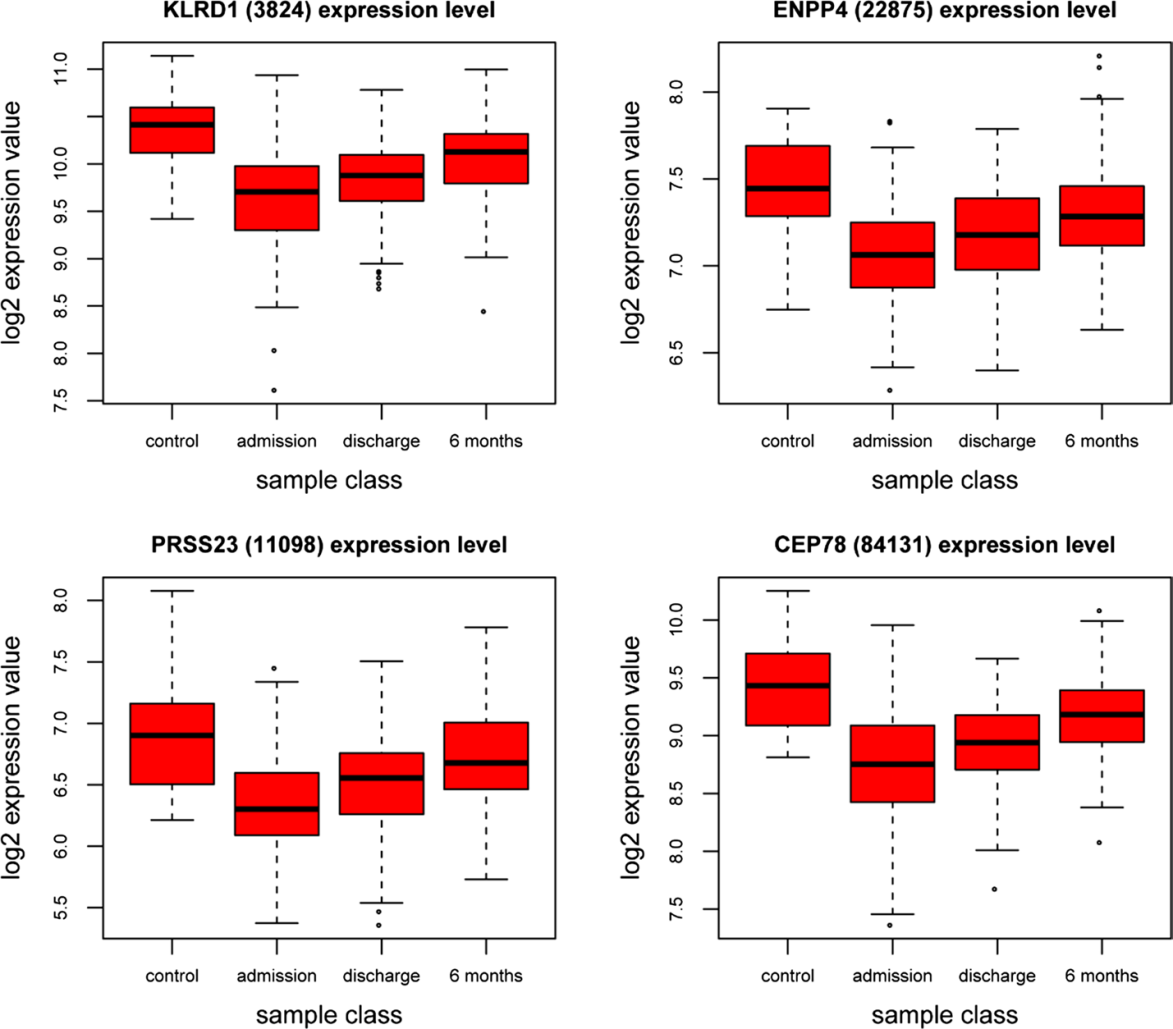
The Expression data from microarray experiments for *KLRD1*, *ENPP4*, *PRSS23*, *CEP78* genes. The y-axis represents the log2 normalized intensity of the gene and the x-axis represents analyzed groups,outlier box plots have been overlaid to show the distribution of the data

The prognosis of patients after acute coronary syndrome (ACS) largely depends on the extent of myocardial damage during the acute phase. In this study, two gene expression profiling in PBMCs—GSE59867 and GSE62646 were downloaded to identify biologically relevant transcripts significantly altered through the AMI follow-up. Next, we performed four comparison: admission versus control, discharge versus control, admission versus 6 month and discharge versus 6 month group, respectively (Fig. 4). Notably, most differentially expressed genes were common in admission versus control and discharge versus control group, with maximum differentially expressed genes in admission versus 6 month and discharge versus 6 month group respectively, which is the same in the pathway analysis (Fig. 5).

These indicating that these gene expression profile modification may be responsible for the functional change during the recovery from AMI. Consistent with pathological changes following ischemic cardiac injury, the top five pathways enriched in the comparison of admission or discharge versus control group were associated with inflammation and immune response, which is also consistent with the previous results in the first section. Interestingly, possibly duo to the pharmacological intervention and myocardial function recovery, the comparison of admission or discharge versus 6 month were functional enriched in metabolic pathways and neurological disease. One possibility is that neurosecretion was involved in a protective systemic response to the development of the cardiac insufficiency in the face of partial or total occlusion of the coronary artery. Previous studies [26] have demonstrated that tyrosine kinase receptor B (TrkB)—a high-affinity receptor for brain-derived neurotrophic factor protects endothelial integrity during atherogenesis and plays a previously unknown protective role in the development of CAD. Also, the long term activation of autonomic nervous system may increase the risk of atherosclerosis, myocardial infarction or sudden death [27, 28].

## Conclusion

In conclusion, the transcriptional profiling presented here demonstrating relationship between inflammation and immune response, Type I diabetes and thyroid disorder and AMI pathological process. On the other hand, we found a set of genes involved in inflammation and immune response on admission and discharge relative to the stable coronary artery disease, and the follow-up of AMI to 6 months associated with the metabolic pathways or neurosecretion. This pilot study warrants further investigation with larger cohorts in the setting of coronary heart disease, it should be very interesting to determine possible interferences in evaluation of diagnostic sensitivity and specificity of the corresponding marker genes, so was the prognostic applicability.
